# Performance and clinical utility of a new supervised machine-learning pipeline in detecting rare ciliopathy patients based on deep phenotyping from electronic health records and semantic similarity

**DOI:** 10.1186/s13023-024-03063-7

**Published:** 2024-02-10

**Authors:** Carole Faviez, Marc Vincent, Nicolas Garcelon, Olivia Boyer, Bertrand Knebelmann, Laurence Heidet, Sophie Saunier, Xiaoyi Chen, Anita Burgun

**Affiliations:** 1 Centre de Recherche des Cordeliers, Université Paris Cité, Sorbonne Université, INSERM UMR 1138, 75006 Paris, France; 2 Inria, 75012 Paris, France; 3 Université Paris Cité, Imagine Institute, Data Science Platform, INSERM UMR 1163, 75015 Paris, France; 4 Department of Pediatric Nephrology, APHP-Centre, Reference Center for Inherited Renal Diseases (MARHEA), Imagine Institute, Hôpital Necker-Enfants Malades, Université Paris Cité, 75015 Paris, France; 5 Laboratory of Renal Hereditary Diseases, INSERM UMR 1163, Imagine Institute, Université Paris Cité, 75015 Paris, France; 6 Nephrology and Transplantation Department, MARHEA, Hôpital Necker-Enfants Malades, AP-HP, Université Paris Cité, 75015 Paris, France; 7 Département d’informatique Médicale, Hôpital Necker-Enfants Malades, AP-HP, 75015 Paris, France

**Keywords:** Diagnosis support, Electronic health record, Supervised machine learning, Semantic similarity, Imbalanced dataset, Rare disease

## Abstract

**Background:**

Rare diseases affect approximately 400 million people worldwide. Many of them suffer from delayed diagnosis. Among them, *NPHP1*-related renal ciliopathies need to be diagnosed as early as possible as potential treatments have been recently investigated with promising results. Our objective was to develop a supervised machine learning pipeline for the detection of *NPHP1* ciliopathy patients from a large number of nephrology patients using electronic health records (EHRs).

**Methods and results:**

We designed a pipeline combining a phenotyping module re-using unstructured EHR data, a semantic similarity module to address the phenotype dependence, a feature selection step to deal with high dimensionality, an undersampling step to address the class imbalance, and a classification step with multiple train-test split for the small number of rare cases. The pipeline was applied to thirty *NPHP1* patients and 7231 controls and achieved good performances (sensitivity 86% with specificity 90%). A qualitative review of the EHRs of 40 misclassified controls showed that 25% had phenotypes belonging to the ciliopathy spectrum, which demonstrates the ability of our system to detect patients with similar conditions.

**Conclusions:**

Our pipeline reached very encouraging performance scores for pre-diagnosing ciliopathy patients. The identified patients could then undergo genetic testing. The same data-driven approach can be adapted to other rare diseases facing underdiagnosis challenges.

**Supplementary Information:**

The online version contains supplementary material available at 10.1186/s13023-024-03063-7.

## Background

Rare diseases affect approximately 400 million people worldwide. Several of them are characterized by extreme clinical and genetic heterogeneity that slows down the process of etiological diagnosis for individual patients. Over the past few years, the increasing digitalization of health-related data brings the opportunity to reuse clinical data to get new insights regarding the management of diseases and to reduce the risks of mis- and delayed diagnosis [1, 2]. One solution to accelerate the diagnosis process is to rely on patients’ electronic health records (EHRs) for automated phenotyping [3] and develop algorithms to match patients’ profiles based on their phenotypes. In other words, EHRs of already diagnosed patients can be reused to suggest an automated phenotype-driven diagnosis for undiagnosed cases [4]. We have shown that clinicians mainly use narrative documents to report on signs, symptoms and comorbidities [5, 6] and that it provided valuable information about the natural histories of rare diseases like Myrhe [7] and Dravet [8, 9] syndromes. Nowadays, several studies use phenotypic information that is present in text and most machine learning models use automated natural language processing (NLP) systems to extract clinically relevant information from EHRs [10], for example for pediatric [11] or kidney diseases [12, 13]. Noaeen et al. [14] confirmed the significance of incorporating NLP-based patient-specific information into prediction models, leading to a notable improvement in the discriminatory power and robustness of the ML algorithms. This is of major importance for rare diseases as early diagnosis can result in better management and progression of the disease [15]. To achieve this goal, the Imagine Institute has developed Dr. Warehouse, a biomedical data warehouse that integrates a Natural Language Processing (NLP) pipeline [16] that enables the extraction of clinical phenotypes from unstructured clinical notes from Necker-Enfants Malades Hospital. We showed that mining Dr. Warehouse accelerated the diagnosis of Dravet syndrome [8] and identified two undiagnosed cases harboring the same de novo variant in *KCNA2* [17]. These two examples demonstrate the utility of this approach to accelerate the diagnosis of difficult cases. Now, our objective is to adapt it to complex and pleiotropic rare diseases such as ciliopathies.

Ciliopathies are an expanding group of more than fifty severe and rare genetic disorders associated with pathogenic variants that result in the abnormal formation or function of cilia. Renal manifestations are common features in ciliopathies [18]. Among renal ciliopathies, recessive nephronophthisis (NPH) is characterized by chronic tubulointerstitial nephritis and the development of massive renal fibrosis and cysts leading to end-stage kidney disease (ESKD), which typically occurs in childhood but also later in adults. NPH can be observed as an isolated condition or as part of multiorgan disorders such as Senior-Løken syndrome or Joubert syndrome. Biallelic variants of the *NPHP1 * gene are the main cause of pediatric NPH. Recent studies have demonstrated that renal ciliopathies are largely underdiagnosed, as high rates of *NPHP1* pathogenic variants (mostly deletion) have been discovered in adults with chronic kidney disease [19] (CKD). Being able to diagnose ciliopathy patients as early as possible is crucial so that they can have access to appropriate support and personalized care and benefit from potential future treatments. Indeed, a potential treatment for *NPHP1*-associated renal ciliopathies has been recently investigated with promising results [20]. Such a treatment might be useful to prevent the onset of severe CKD for patients at an early stage but is also expected to slow down the evolution of CKD for patients with kidney lesions. Diagnosing patients regardless of the CKD severity is also important for patients’ families so that their relatives can be given molecular diagnosis at an early stage through cascade screening.

In a previous study [21], we used word embeddings and a similarity-based ranking model to identify from the EHRs the top-ranked patients who were most likely to have any ciliopathy disorder. We obtained good precision scores but a very low sensitivity. Based on these results and considering the recent promising therapeutic advances for *NPHP1* conditions, we decided to design a machine-learning based algorithm focusing on *NPHP1* cases.

Detecting rare patients within large hospital EHR repositories is challenging for at least three reasons: the high dimensionality of data, the important imbalance between cases (the minority class) and controls (the majority class), and the dependence between phenotypes.

To address the high dimensionality issue, some feature selection processes can be applied to raw data [22]. It can rely on expert knowledge, or it can be an automated task trained to select the most relevant and nonredundant features for a specific classification task [22, 23]. Regarding imbalance, data-based methods, such as undersampling and oversampling, are often considered to balance the two classes.

To consider the dependence between phenotypes, the semantic similarity between two phenotypes may be calculated based on their relative position in a hierarchy and the information content (IC) of their most informative common ancestor (MICA) [24, 25]. An alternative consists of using phenotype embeddings, which are vector representations that capture word context in a document and encode its meaning such that concepts with similar meanings are closer in the vector space. However, these two methods were usually implemented as an isolated approach and were rarely considered in a machine learning context.

Our objective is to develop a supervised machine learning pipeline based on automatic phenotyping from unstructured EHRs to identify undiagnosed patients bearing *NPHP1* variants while addressing the three aforementioned challenges. The patients classified as potentially having *NPHP1* ciliopathy could then undergo genetic testing for diagnosis confirmation and be eligible for potential future treatment. This study was performed in the context of the C’IL-LICO project, a research initiative coordinated by the Imagine Institute that aims to accelerate the diagnosis and treatment of ciliopathy patients.

## Methods

### Databases and data encoding

The Necker-Enfants Malades Hospital is a French reference center for rare and undiagnosed diseases that belongs to the Greater Paris Hospital (AP-HP) and hosts the Imagine Institute, a research center specializing in genetic diseases. Its clinical data warehouse, Dr. Warehouse [16] contains EHRs of more than 800,000 patients, including more than 9 million clinical documents, which constitute a major source of clinical signs and phenotypes [26]. The high-throughput phenotyping module of Dr. Warehouse enables the automatic extraction of all types of clinical entities from EHRs based on the UMLS Metathesaurus^®^ [27]. Besides detection and normalization, the module performs disambiguation, negation and subject prediction [28], which are fundamental to determine whether a clinical sign is present or absent, experienced by the patient or by their relatives. In this analysis, we focused on present phenotypes experienced by the patient. As the hierarchy of the Human Phenotype Ontology (HPO), a standard vocabulary for rare disease phenotypes used in clinical practice and genetic research, looked more appropriate than the UMLS network for computing semantic similarity, we converted all extracted UMLS phenotypes to HPO concepts based on the mapping provided by the HPO consortium (HPO format-version: 1.2; data-version: releases/2020-12-07; downloaded on 2020-12-10).

As a reference for ciliopathy cases, we used a research database named Cilio-base, developed at the Imagine Institute that contains structured research information for more than 1800 patients clinically diagnosed with ciliopathies, including diagnoses and causal genes. 1103 of the patients have biallelic variants in one causative gene identified. 116 different causative genes were identified in these patients. In the present study, we focused on *NPHP1* ciliopathy cases followed at Necker Hospital (i.e., who have EHRs in Dr. Warehouse). Of note, Cilio-base does not contain patients diagnosed with autosomal dominant polycystic kidney disease (ADPKD) or autosomal recessive polycystic kidney disease (ARPKD) because these ciliopathy subtypes are well described and easier to diagnose.

### Patient selection and extraction of phenotypes

We included as cases all patients with ciliopathy caused by *NPHP1* biallelic pathogenic variants who were followed at Necker Hospital.

The main symptom associated with *NPHP1* variants is the degradation of kidney function, which leads to ESKD, and consequently dialysis and/or transplantation. As our objective was to diagnose patients as early as possible and because post-transplantation events can provide noisy phenotypes, we decided to use only the phenotypes that occurred before the transplantation. The transplantation date was manually checked for all ciliopathy cases.

To distinguish between patients with *NPHP1* ciliopathy and patients with other kidney diseases, we defined control patients as patients who exhibited some overlapping phenotypes with *NPHP1* ciliopathy patients, namely, kidney defects. We thus randomly selected from Dr. Warehouse 10,000 nonciliopathy patients (i.e., excluding patients who were present in Cilio-base) having at least one automatically extracted UMLS concept subsumed by *Kidney diseases* ([C0022658]) or by *Congenital anomaly of the kidney* ([C0266292]) in their EHRs. The number of controls was determined to emulate the critical imbalance that exists in real-life conditions. Afterwards, control patients who had undergone a kidney transplantation were automatically excluded to avoid phenotypes associated with post-transplantation events.

**Fig. 1:**
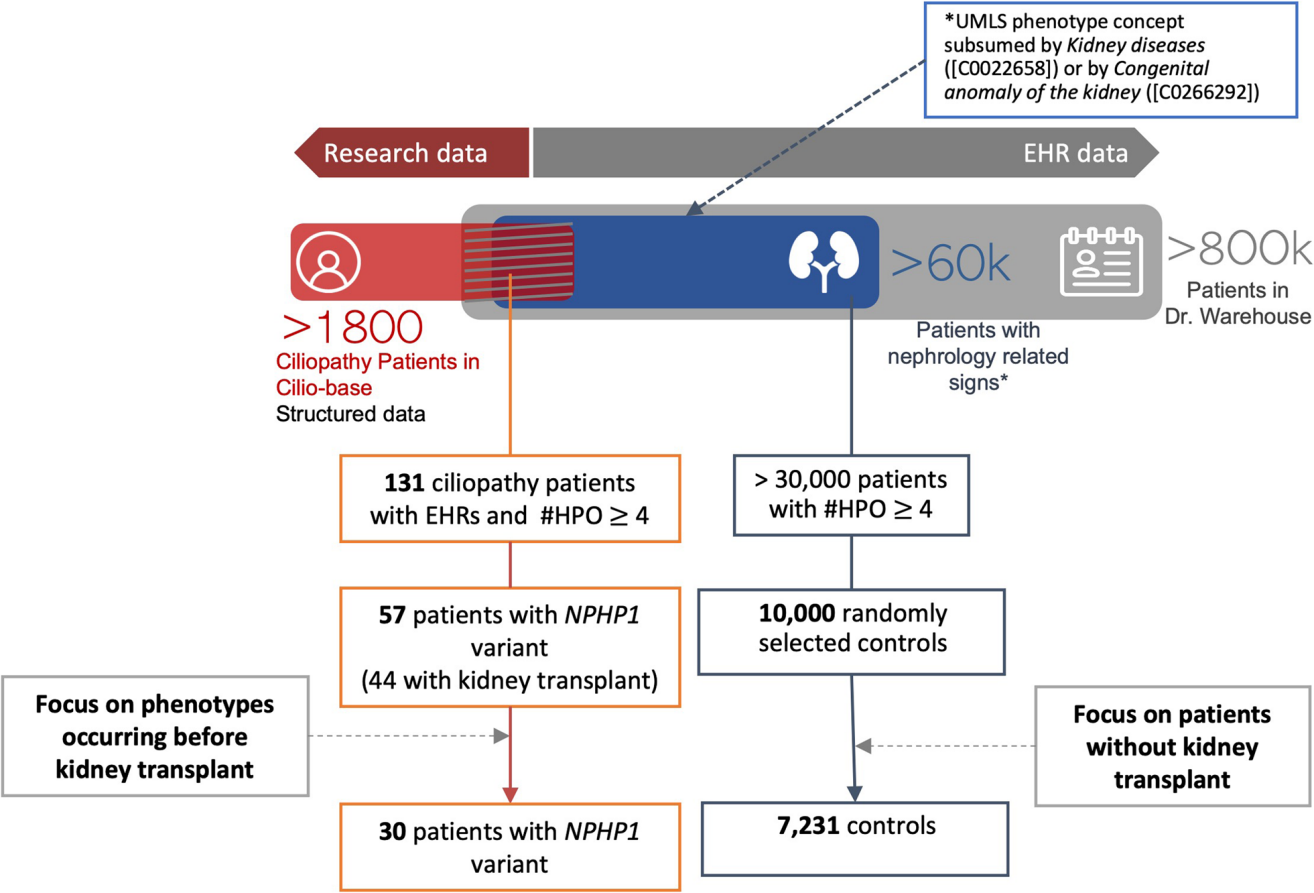
Flowchart of the patient selection process

For cases and controls, phenotypes were extracted from clinical notes using the phenotyping module of Dr Warehouse and converted from UMLS to HPO. To ensure that sufficient phenotypic information was available, we limited ourselves to cases and controls with at least four distinct HPO concepts extracted from their EHRs (Fig. 1).

### Semantic similarity based on HPO

We first considered the method published by Lin [25] to compute the similarity between HPO concepts. We chose this classic similarity-based method after performing some preliminary tests that showed very few differences in performance among the different methods. This finding is similar to the one of Jia et al. who concluded in their study on patient similarity measurement that “IC-based code-level similarity algorithms are comparable to each other” [29, 30]. This method starts by determining the IC of each phenotype p in the HPO, defined as $IC\left(p\right)= -log(\frac{n}{N})$, with $n$ the number of descendants of $p$ and $N$ the maximum number of concepts in the HPO. The similarity between two phenotypes ${p}_{1}$ and ${p}_{2}$ is calculated based on the IC of their MICA in the HPO:

1 $$Sim_{Lin} (p_{1,} p_{2} ) = \frac{{2 \times IC\left( {MICA} \right)}}{{IC\left( {p_{1} } \right) + IC\left( {p_{2} } \right)}}$$

One concern with this method is that two terms can be close in a hierarchy but have completely different mechanisms and etiologies, such as CKD and acute kidney injury (AKI). Consequently, we proposed an adjustment of the Lin method where the similarity between two terms is set to 0 if there is no subsumption relationship between them:

2 $$Sim_{RestrLin} (p_{1} ,p_{2} ) = \left\{ {\begin{array}{*{20}l} {\frac{{2 \times IC\left( {MICA} \right)}}{{IC\left( {p_{1} } \right) + IC\left( {p_{2} } \right)}}} \hfill & { if\, p_{1} \,and\, p_{2} \,on\, the \,same\, branch \,of\, the\, hierarchy } \hfill \\ {0 } \hfill & { otherwize. } \hfill \\ \end{array} } \right.$$

The similarity metrics *Sim*_*Lin*_ and *Sim*_*RestrLin*_ are referred to as Lin similarity and restricted hierarchical similarity, respectively.

### Semantic similarity with term embeddings

We considered two embedding methods: (1) fastText embeddings trained locally on 2.5 million documents from Necker [28], and (2) CODER embeddings [31], which are medical term embeddings infused with relational knowledge from UMLS showing good performance in various NLP tasks, outperforming other state-of-the-art biomedical word embeddings. In both cases, the semantic similarity between two phenotypes ${p}_{1}$ and ${p}_{2}$ is calculated using cosine similarity between two embeddings denoted as

3 $$Sim_{cos} \left( {p_{1} , p_{2} } \right)$$

These methods are referred to as fastText embeddings and CODER embeddings, respectively.

### Patient representation

Let $P$ denote the set of distinct phenotypes in our dataset, and let $D$ denote the set of phenotypes present for a patient. Each patient is represented as a vector $V$ with length($V$) = Card(P). In the baseline approach, $V$ is filled with binary values corresponding to the presence or absence of the phenotype ${p}_{i}$ for the patient. In the semantic cases, the vector $V$ is filled with the maximum similarity between the phenotype ${p}_{i}$ and the set of phenotypes D present for the patient:

$$V\left[ i \right] = Max \left\{ {d_{j} \in D} \right\} Sim\left( {p_{i} ,d_{j} } \right)$$

### Preprocessing and classification

To address the high dimensionality of the data, automatic feature selection using a Random Forest Classifier was applied as a preprocessing step, to keep only a subset of relevant phenotypes.

Regarding the imbalance between classes, we used an undersampling technique, Cluster Centroids, which selects a representative subset of controls based on the k-means algorithm.

Four classification methods, namely, a linear model, ridge regression (RidgeReg), a non-linear model, support vector machine (SVM), and two ensemble models, random forests (RF) and Gradient boosting (XGBoost), were considered. Gradient boosting is a well-established variant of ensemble learning methods, i.e., methods that combine many models together to create a final model. XGBoost has outperformed numerous machine learning models in recent challenges [32, 33]. We trained our models using fivefold cross-validation on two-thirds of the data and tested them on the remaining one-third.

For each classification method combined with the preprocessing steps (feature selection + undersampling), the hyperparameter settings reaching the best average performance within the cross-validation on the training set were used to predict the patient class on the test set (not undersampled). The range of hyperparameters tested for each classifier is given as supplementary Methods (see Additional file 1). As the number of cases was low, the whole process was repeated 10 times to increase the reliability.

### Evaluation

**Fig. 2:**
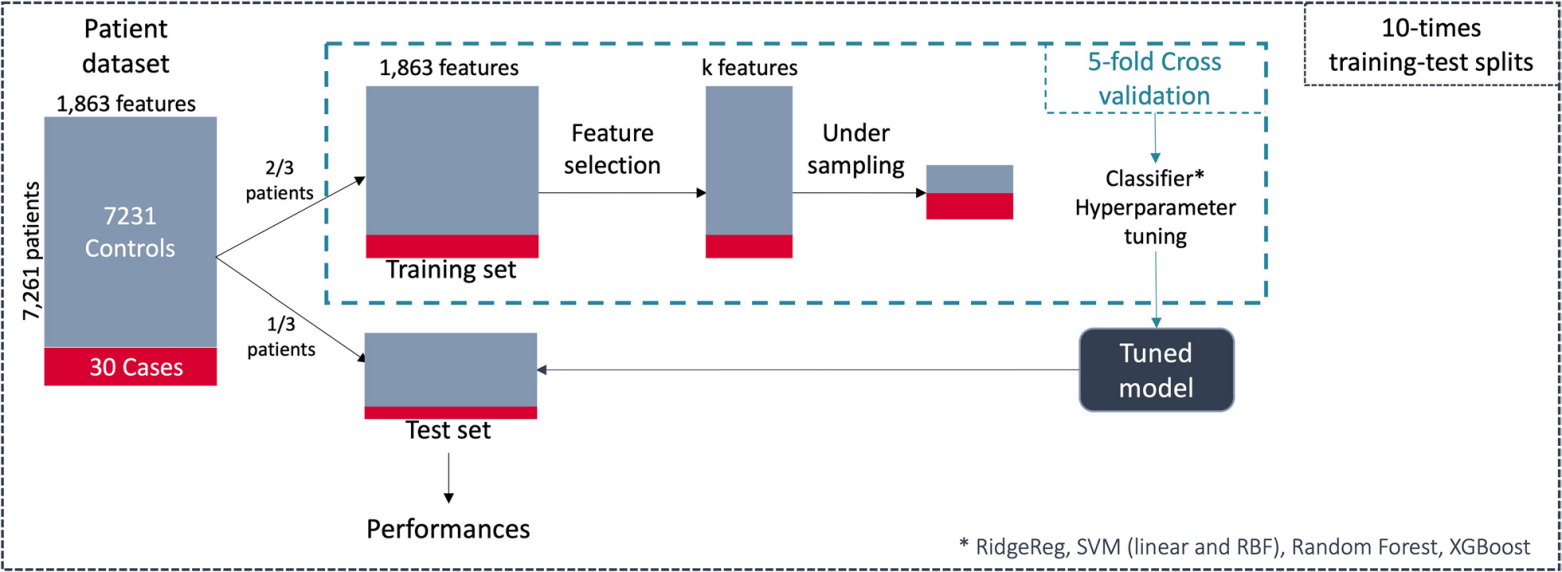
Flowchart of the evaluation process

Regarding performance metrics, to be able to compare the performances of the different algorithms and to get consistent and interpretable results, we fixed the specificity to 90% by tuning the decision thresholds of the different algorithms and we focused on the sensitivity. In addition, the predictive performance was evaluated by assessing the number of cases within the set of patients identified by the classifier as at highest risk of having ciliopathy: all patients were ranked using their class probability, then sensitivity (also called recall) and precision (also called predictive positive value) among the top 1% and 10% highest ranked patients were provided, which were denoted as recall@k% and precision@k%, respectively. We also provided metrics of the global performances of the classifiers, such as the area under the receiver operating curve (AUROC) and under the precision-recall curve (AUPRC), which is generally more suited for imbalanced datasets. AUPRC must be interpreted in the context of imbalanced data, as the value is strongly impacted by data imbalance. The overall evaluation process is summarized in Fig. 2.

### Implementation

Analyses were performed with Python version 3.8 using scikit-learn, imbalanced-learn, UMAP, statistics, matplotlib packages, and R version 3.6.3 using tidyverse, OntologyIndex, OntologySimilarity packages.

## Results

### Characteristics of the patients

Fifty-seven ciliopathy patients with *NPHP1* biallelic pathogenic variants were followed at Necker Hospital with sufficient phenotypic information. Twenty-seven cases were excluded because these patients were hospitalized at our center exclusively to receive a kidney transplant and consequently had no phenotypes in their EHR before. Among the 30 remaining cases, 17 patients underwent a kidney transplantation, for whom only the phenotypes collected before the procedure were kept. Regarding diagnosis, 18 patients were diagnosed with an isolated form of NPH, 8 patients had NPH and neurological symptoms (3 patients with Joubert syndrome, 5 with other brain defects), and 4 patients were diagnosed with Senior-Løken syndrome (renal and ocular defects). Regarding the CKD severity, at the date of the most recent captured phenotype, 18 (60%) patients had not reached ESKD yet (2 patients with mild kidney damage and 16 patients with moderate to severe kidney damage), and 12 patients (40%) had reached ESKD. As mentioned in the Introduction, diagnosing these ESKD patients is less crucial although still very important to avoid diagnosing wandering.

A total of 10,000 controls with kidney-related symptoms and sufficient phenotypic information were randomly selected, among which 2769 patients with kidney transplants were removed.

The final dataset consisted of 30 ciliopathy cases and 7,231 controls (Fig. 1). Patient characteristics are displayed in Table 1. Regarding extracted HPO concepts (Table 2), general renal defects (e.g., renal insufficiency, abnormality of the kidney) were strongly represented among both cases and controls. HPO concepts that can be indicative of ciliopathy related CKD were more frequent among cases (e.g., anemia, hyperparathyroidism, polydipsia, enuresis).

**Table 1:** Patient characteristics

	Ciliopathy cases	Controls
# Training set	20	4844
# Test set	10	2387
# Total	30	7231
Sex ratio (M/F)	1.5	1
Age* (median (IQR))	14.8 (12–19.2)	11.5 (4.6–25.8)
% Syndromic forms	40%	NA
#HPO (median (IQR))	18 (10.3–35.8)	10 (6–18)

^*^For each patient, age corresponds to the age at the date of the most recent EHR document. # Number of patients

**Table 2:** Top 20 HPO concepts among cases and controls

Ciliopathy cases (n = 30)		Controls (n = 7231)	
HPO concepts	# Patients (%)	HPO concepts	# Patients (%)
Chronic kidney disease	77	Fever	47
Abnormality of the kidney	73	Pyelonephritis	32
Nephropathy	73	Proteinuria	31
Renal insufficiency	73	Pain	26
Anemia	53	Seizure	17
Fatigue	53	Renal insufficiency	15
Proteinuria	53	Vesicoureteral reflux	15
Arteriovenous fistula	47	Muscular hypotonia	15
Stage 5 chronic kidney disease	43	Cough	14
Hypertension	40	Diarrhea	14
Hyperparathyroidism	37	Hematuria	13
Pain	37	Constipation	12
Pollakisuria	37	Abnormality of the kidney	12
Anorexia	30	Increased body weight	12
Fever	30	Hypertension	12
Nausea	30	Moderate albuminuria	11
Polydipsia	30	Edema	11
Enuresis	27	Albuminuria	11
Hypokalemia	27	Nephropathy	11
Moderate albuminuria	27	Respiratory distress	11

# Number of patients

### Performance of the DSS

A total of 7,261 patients (*NPHP1* + controls) presented 1,863 distinct phenotypes (Fig. 2). In each train-test split, 20 cases and 4,844 controls were used to train the model. At each round of the cross-validation, between 250 and 600 phenotypes were automatically kept after the feature selection, depending on the similarity method used. Regardless of the method considered, the selected features included phenotypes known to be associated with ciliopathy, such as CKD, polydipsia, and molar tooth sign on the MRI, as well as phenotypes such as AKI, which allows distinguishing between ciliopathy patients and controls.

The best hyperparameters were determined via grid search and then applied to the test set. The performance metrics were computed in the test set, then averaged across the 10 repetitions of train-test split for each classifier combined with each method of semantic similarity (Table 3).

**Table 3:** Performance of each classifier combined with each semantic similarity method in the test set

Similarity	Method	Mean sens.* (IC 95%)	Recall @1% (%)	Recall @10% (%)	Precision @1% (%)	Precision @10% (%)	Mean AUROC (IC 95%)	Mean AUPRC (IC 95%)
Baseline	RidgeReg	82% [76–88]	**59**	81	**25**	3.4	93% [91–95]	**46% [36–56]**
	SVM	81% [75–87]	54	81	23	3.4	91% [89–93]	**45% [36–54] **
	RF	78% [73–83]	55	78	23	3.3	91% [89–93]	**47% [39–55]**
	XGBoost	82% [76–88]	**61**	82	**25**	3.4	93% [91–95]	**42% [33–51] **
Lin similarity	RidgeReg	65% [57–73]	23	65	10	2.7	85% [82–88]	6.0% [4.0–8.0]
	SVM	68% [60–76]	32	62	13	2.6	84% [81–87]	15% [9–21]
	RF	76% [68–84]	44	76	18	3.2	90% [87–93]	21% [13–29]
	XGBoost	63% [51–75]	18	62	8	2.6	85% [81–89]	6.0% [3.0–9.0]
Restricted hier. sim	RidgeReg	76% [71–81]	49	76	20	3.2	92% [90–94]	30% [21–39]
	SVM	71% [54–88]	43	72	18	3.0	84% [72–96]	31% [23–39]
	RF	**85% [79–91]**	**59**	**85**	**25**	**3.5**	93% [90–96]	**43% [35–51]**
	XGBoost	**86% [80–92]**	41	**86**	17	**3.6**	**96% [94–98]**	35% [23–47]
fastText Embd	RidgeReg	81% [76–86]	48	79	20	3.3	90% [86–94]	35% [25–45]
	SVM	81% [76–86]	52	79	22	3.3	91% [88–94]	33% [23–43]
	RF	76% [70–82]	50	77	21	3.2	89% [86–92]	30% [20–40]
	XGBoost	77% [70–84]	38	77	16	3.2	88% [83–93]	19% [13–25]
CODER Embd	RidgeReg	**84% [78–90]**	57	**86**	24	**3.6**	91% [88–94]	35% [25–45]
	SVM	78% [72–84]	53	77	22	3.2	89% [86–92]	40% [30–50]
	RF	74% [68–80]	48	74	20	3.1	88% [85–91]	35% [23–47]
	XGBoost	72% [66–78]	39	72	16	3.0	87% [83–91]	19% [12–26]

The bold values indicate high performance

*RF* random forest, *SVM* support vector machine, *Sens.* sensitivity

^*^Sensitivity for a specificity of 90%

Within our pipeline (filtering based on kidney transplantation, feature selection), the baseline method already achieved high performances with all classifiers reaching sensitivity scores around 80% for a 90% specificity. AUROC [0.91–0.95] and AUPRC [0.44–0.47] were high considering our imbalanced context, as the AUPRC is expected to be 0.0041 using a random model (based on the prevalence of the cases in the dataset). The baseline model also showed a strong ability in identifying patients who were most likely to have ciliopathy since the recall@1% was between [54–61%]. Using Lin similarity decreased the sensitivity compared to the baseline method. However, the restriction to ascendants-descendants successfully addressed this issue and provided higher performances as expected. The restricted hierarchical similarity combined with ensemble methods (RF and XGBoost) reached overall the highest AUROC and highest sensitivity, demonstrating that the restricted hierarchical similarity was able to prevent the misclassification of control patients presenting phenotypes that were siblings of ciliopathy-related phenotypes. This can be illustrated with CKD versus AKI: while Lin similarity considers these two phenotypes as highly similar, the restricted hierarchical similarity between these two phenotypes is null, leading to fewer false positives and better overall performance. Considering the top ranked patients, restricted hierarchical similarity with RF showed a strong capacity to identify ciliopathy patients, with a recall@1% of 59% (i.e., precision around 25% whereas using a random model it would be 0.41%) and a recall@10% of 85%. Comparable performances among top 1% and top 10% were obtained by CODER embeddings with ridge regression. Embedding methods reached overall high performances, especially with non-ensemble methods.

### Qualitative analysis

To get qualitative insights regarding the performance, we performed a manual review of the EHRs of some patients misclassified by the restricted hierarchical model with RF. We focused on this method because it achieved strong overall performances (AUROC = 0.93, AUPRC = 0.43) and high ability to mitigate the number of false positives among the top ranked patients (sensitivity = 85%, recall@1% = 59%, recall@10% = 85%). Over the 10 runs, five ciliopathy patients were systematically classified as controls by this method (false negatives). These patients were also often classified as controls by other methods. Four of them (P1, P3, P4, P5) had isolated NPH, and one patient (P2) had renal and brain defects. Despite the restriction to patients with at least four distinct HPO concepts, the review of these five patient profiles showed that their EHRs contained poor phenotypic descriptions regarding ciliopathy:

- Three patients (P1–P3) were followed up in other hospitals and had only one document in Dr. Warehouse.
- One patient (P4) underwent a kidney transplantation, and his only visit to Necker before that was for heart surgery. Consequently, he had no ciliopathy-related phenotype recorded in his EHR before transplantation.
- The last patient (P5) had only a few extracted ciliopathy-related symptoms.

Notably, *NPHP1* molecular diagnosis in P3 and P5 was based on family cascade screening, but these patients exhibited no (P5) or very mild (P3) symptoms upon genetic testing. Moreover, four patients (P1–3 and P5) had symptoms compatible with *NPHP1* ciliopathy related CKD such as “reins discrètement hyperéchogènes” (mildly hyperechogenic kidneys), “infléchissement staturo-pondéral” (growth faltering) or “syndrome polyuro-polydipsique” (polyuro-polydipsia syndrome), that the UMLS-based extraction module of Dr. Warehouse failed to extract, because the expression used in the documents did not exist in the UMLS.

**Fig. 3:**
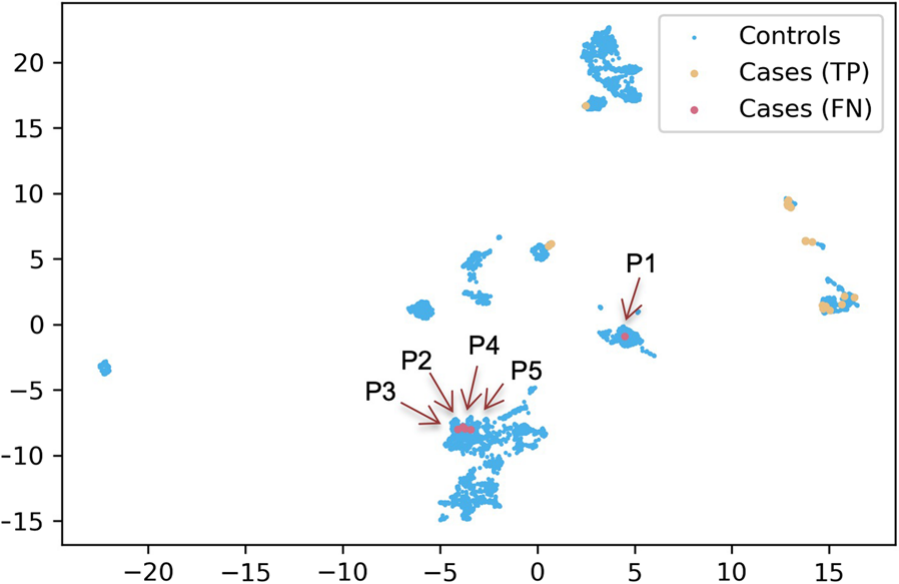
UMAP with restricted hierarchical similarity. *TP* true positives; *FN* false negatives, i.e., cases always misclassified by the method, corresponding to patients P1–5

Interestingly, when performing uniform manifold approximation and projection (UMAP) using restricted hierarchical similarity (Fig. 3), four patients (P2–5) were grouped, whereas P1 was isolated in a cluster of controls containing many false positives. Presence of P1 in the training set may explain the number of false positives with common renal defects, which highlights the role of data quality especially in case of “small data”.

Regarding false positives, a ciliopathy expert (SS) reviewed the EHRs of 40 control patients who were classified as having ciliopathies when using the default decision threshold and were grouped with true positives with UMAP. Among them, ten patients (25%) had phenotypes belonging to the ciliopathy spectrum:

- Five patients were diagnosed with ADPKD (3 confirmed and 2 strongly suspected), and one patient was diagnosed with ARPKD. As explained in section “Databases and data encoding”, these patients were not included in Cilio-base. Consequently, some of them were present in our control dataset.
- One patient had been suspected by clinicians to have NPH but then confirmed otherwise.
- Three patients were suspected to have a pathogenic variant in the *HNF1B* gene, which is known to potentially impact ciliary function and to lead to overlapping phenotypes with ciliopathy related CKD.

Thus, our classification model classified as ciliopathy some controls who had phenotypes belonging to the ciliopathy spectrum, which is a good indicator that the pipeline can detect patients that could be suspected to have ciliopathy.

Other recurrent diagnoses included glomerulopathies (7 patients), infection-induced kidney disease (3 patients), AKI (3 patients), and chronic tubulointerstitial nephritis due to lithium administration (2 patients), which is a differential diagnosis for NPH.

## Discussion

### Main results

With the objective of distinguishing between *NPHP1*-mutated patients and other nephropathies, we designed a pipeline that considers the problem as a data-driven classification task. The supervised machine learning pipeline combines a phenotyping module re-using unstructured EHR data, semantic similarity measures to integrate dependence among phenotypes, a feature selection step to deal with the high dimensionality, an undersampling step to address the data imbalance, and a classification step with multiple train-test split for the small number of NPH cases.

The performances were generally high, especially when using the baseline model, the restricted hierarchical similarity or CODER embeddings. This is an encouraging result, given that 60% of *NPHP1* patients have isolated NPH. Of note, the linear models achieved good results, and the general performances were not systematically improved using more complex models, as already observed by other authors [26, 34, 35]. Although the best AUPRC were obtained with the baseline model, the restricted hierarchical similarity reached high overall performances and the best sensitivity for a 90% specificity. Such high true positive rates are essential to ensure that the number of patients classified as ciliopathy, i.e., who would be referred to a geneticist, can be managed in real life.

Regarding other attempts to detect rare diseases patients among large-scale clinical databases, very few systems have been developed yet relying on machine learning and phenotypes. We identified seven articles corresponding to these criteria [34–40], all published between 2020 and 2022. When provided, the specificity reached by these systems was high [85–99%], whereas the sensitivity was generally low, ranging from 14 to 64% when data imbalance was considered.

### Technical significance

Our approach differs from the disease recommendation systems usually used in the rare disease domain [4]. Indeed, disease recommendation systems can also rely on disease similarity [41, 42]. They generally work as follows: each rare disease is described by a set of phenotype concepts usually extracted from knowledge bases and the system returns a list of diseases ranked by the score of similarity between the patient and a rare disease. However, their objective is to provide a diagnosis for any undiagnosed rare disease patient. In this study, we investigated a different approach, which is to integrate phenotypic similarity into the patient vectorial representation and then classify patients using a machine learning predictive model. Such a data-driven approach aims at identifying patients with *NPHP1* ciliopathy from large-scale clinical databases containing both rare and common diseases.

Our pipeline reached very good performance despite a very low number of *NPHP1*-mutated patients. Contrasting with the poor results obtained without undersampling, very high specificity was reached with ClusterCentroids undersampling, although only 0.41% of controls were used to train the model, showing that ClusterCentroids is a good strategy to minimize underfitting.

Contrasting with other approaches [35, 38–40], in our work, we minimized the integration of a priori expert knowledge. As knowledge regarding ciliopathies is still evolving, and as our objective is to design a pipeline that can be easily adapted to other rare diseases suffering from underdiagnosis, we decided to focus on a data-driven approach.

Regarding the similarity calculation, we think that the similarity between two phenotypes should integrate some physio-pathological features. Grani et al. [43], for example, stated that disease ontologies and taxonomies should integrate more etiology and genetic causes of diseases. The mitigated results obtained by the classic Lin similarity show the need to apply similarity methods on appropriate ontologies or to use methods where the proximity between concepts would reflect common mechanisms. Here, we demonstrated that similarity restricted to ascendant-descendant relations was more relevant than the basic Lin approach. Chen et al. reached a similar conclusion for developing a disease-ranking model for rare disease diagnoses [44]. However, we think further work is needed to improve the modeling of the dependence and relatedness between phenotypes. Methods based on node embeddings for example [45] could be interesting to test.

While most models trained to predict diagnosis have used only structured data until now [26, 34], it has been showed that text reports provide much more phenotypic information [15, 46, 47]. Of note, we did not process raw data such as images and measurements. However, we took all medical notes from the patients’ EHRs, which include all types of reports, such as radiology or laboratory test reports. All abnormal results are usually transcribed as phenotypes in the text of these reports (e.g., “renal cyst” or “small kidney” in the MRI report) and interpreted by the clinicians in their notes (e.g., translated into “proteinuria” or “anemia”). However, it is difficult to obtain complete patient representations due to poor-quality data, incompleteness or heterogeneity [47, 48]. The manual review of the false negatives showed that patients with very few visits—or even not followed at Necker Hospital—had phenotypic descriptions that were so poor that it was impossible to automatically categorize them as *NPHP1*-case. Moreover, some of them had ciliopathy-related phenotypes that were not captured by our thesaurus-based extraction pipelines. This issue is of major importance for rare diseases when the number of cases is very small and high-quality data is needed to train the model. Named Entity Recognition approaches such as PhenoBERT [49] or hybrid methods to link textual mentions to concepts in an ontology [50] obtained encouraging preliminary results. In another article [51], we showed how we strongly improved the automatic extraction of phenotypes from EHRs for a ciliopathy named Jeune syndrome using a hybrid method combining a dictionary-based method with deep learning. We plan to adapt this method [51] for *NPHP1* patients and evaluate whether it can further improve patient classification. We expect to obtain an even better performance of our diagnosis algorithm with a more comprehensive set of automatically extracted phenotypes.

### Clinical significance

According to König et al. [52], 63% of patients with *NPHP1* variants reach ESKD at a mean age of 11.4. Recently, a group of researchers from the Imagine Institute identified a potential first-class treatment for *NPHP1*-associated renal ciliopathies [3]. Patients at risk of carrying this variant must be diagnosed without delay to have access to appropriate support and personalized care and to benefit from potential future treatments. Moreover, a recent study published by Snoek et al. [19] on more than 5000 ESKD patients showed that *NPHP1* was a relatively frequent monogenic cause of adult-onset ESKD but that most patients with *NPHP1* variants were given a diagnosis of “chronic kidney disease with unknown etiology” [19]. We trained and validated our system on a population from a children’s hospital, where the mean age was around 17 years old for ciliopathy patients and controls, and we plan to extend its coverage to adult nephropathy patients soon.

Another interesting point raised in this study is the high percentage (25%) of patients with phenotypes belonging to the ciliopathy spectrum in the set of patients considered “false positives”. This result demonstrates the ability of the system to find highly similar patients and the interest in applying this approach to domains where the distinction between cases and controls is still rather fuzzy.

### Perspectives

We plan to conduct further investigations and tests on data from non-specialized hospitals (children and adults), where the probability to find undiagnosed *NPHP1* patients is much higher than in reference centers for rare and undiagnosed diseases like Necker Hospital.

As previously stated, in our work, we minimized the integration of a priori expert knowledge; our approach is fully automated and relies on phenotypes and not on specific disease-related material and can be easily adapted to other rare diseases suffering from underdiagnosis. One of our perspectives is now to train and evaluate this current system on other ciliopathy patients. The next step will be to evaluate the impact of enriching our pipeline with extra information such as demographics, negative phenotypes [53, 54], and lab test results on the classification performance. We will evaluate it within a two-step pipeline, where such kind of information is used as a second step to improve the differential diagnosis process.

## Conclusion

We developed a supervised machine learning pipeline based on deep phenotyping of EHRs that can help improve the detection of patients with rare and complex diseases such as ciliopathies. Relying on semantic similarity improved the performance, especially regarding the sensitivity. We think the lessons learned from this analysis can be of interest for other research teams working on rare disease diagnosis. The next steps will consist of testing our method on adult datasets and other hospitals and extending our data-driven approach further to other rare diseases.

### Supplementary Information

**Additional file 1**. Supplementary method describing the range of hyperparameters tested for each classifier.

## Data Availability

The clinical datasets from this study are not publicly available, as institutional officials expressed concern about the inability to guarantee anonymity. Aggregate data and datasets containing coarse-grained phenotypes are available upon request to the corresponding author.
