# Supplementary material for: Performance and clinical utility of a new supervised machine-learning pipeline in detecting rare ciliopathy patients based on deep phenotyping from electronic health records and semantic similarity
Source: Orphanet J Rare Dis. 2024 Feb 10;19:55. doi: 10.1186/s13023-024-03063-7 (PMC10858490; doi:10.1186/s13023-024-03063-7)
Supplement: Supplementary file 1 — Additional file 1. Supplementary method describing the range of hyperparameters tested for each classifier. [file 13023_2024_3063_MOESM1_ESM.docx]

**Hyperparameter tuning**

A range of hyperparameters was selected for each classifier, i.e., the regularization strength (0.5, 1, 5, or 10) for ridge regression; the kernel type (linear or RBF), the regularization parameter (1, 5, 10, or 100) and the kernel coefficient (0.0001, 0.001, 0.01, or 0.1) for SVM; the maximum depth (3, 5, or 8) and the number of estimators/trees (10, 100, or 1000) for random forests; the maximum depth (3, 5 or 8), the minimum child weight (1, or 3), the learning rate (0.01, 0.1, 0.3) and the feature subsample ratio (0.3, 0.6, 1) for XGBoost. The best hyperparameters were determined via grid search and then applied to the test set.
